# Applications of artificial intelligence in computed tomography imaging for phenotyping pulmonary hypertension

**DOI:** 10.1097/MCP.0000000000001103

**Published:** 2024-07-09

**Authors:** Michael J. Sharkey, Elliot W. Checkley, Andrew J. Swift

**Affiliations:** a Department of Clinical Medicine, University of Sheffield; b 3D Imaging Lab, Sheffield Teaching Hospitals NHS Foundation Trust; c Insigneo Institute for in Silico Medicine, University of Sheffield; d National Institute for Health and Care Research, Sheffield Biomedical Research Centre, Sheffield, UK

**Keywords:** artificial intelligence, computer tomography, pulmonary hypertension

## Abstract

**Purpose of review:**

Pulmonary hypertension is a heterogeneous condition with significant morbidity and mortality. Computer tomography (CT) plays a central role in determining the phenotype of pulmonary hypertension, informing treatment strategies. Many artificial intelligence tools have been developed in this modality for the assessment of pulmonary hypertension. This article reviews the latest CT artificial intelligence applications in pulmonary hypertension and related diseases.

**Recent findings:**

Multistructure segmentation tools have been developed in both pulmonary hypertension and nonpulmonary hypertension cohorts using state-of-the-art UNet architecture. These segmentations correspond well with those of trained radiologists, giving clinically valuable metrics in significantly less time. Artificial intelligence lung parenchymal assessment accurately identifies and quantifies lung disease patterns by integrating multiple radiomic techniques such as texture analysis and classification. This gives valuable information on disease burden and prognosis. There are many accurate artificial intelligence tools to detect acute pulmonary embolism. Detection of chronic pulmonary embolism proves more challenging with further research required.

**Summary:**

There are numerous artificial intelligence tools being developed to identify and quantify many clinically relevant parameters in both pulmonary hypertension and related disease cohorts. These potentially provide accurate and efficient clinical information, impacting clinical decision-making.

## INTRODUCTION

Pulmonary hypertension is a chronic condition defined by elevated mean pulmonary arterial pressure (mPAP) at rest. Pulmonary hypertension is estimated to affect approximately 1% of the global population, causing significant morbidity and mortality. Pulmonary hypertension is a heterogeneous condition, accurate patient phenotyping is essential to determine the cause and subsequent therapeutic approach. Diagnosis is made based on multiple investigations; biochemical, lung function, radiological and invasive investigations. This determines the cause of the condition and informs ongoing management [1^▪▪^]. Computer tomography (CT) plays a central role in determining the phenotype of each patient. Many artificial intelligence techniques have been developed in this modality to aid in the phenotyping of pulmonary hypertension. This article reviews the latest CT artificial intelligence applications in pulmonary hypertension and related diseases. 

**Box 1:**
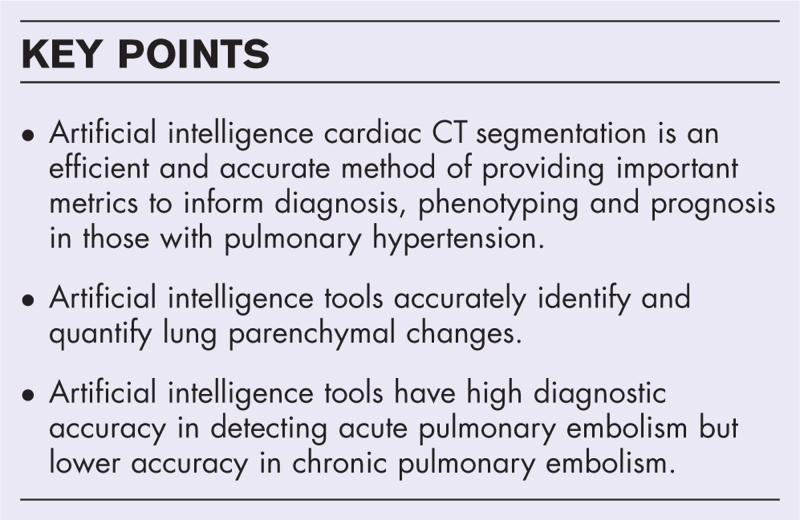
no caption available

## PULMONARY HYPERTENSION

Pulmonary hypertension is defined as an mPAP greater than 20 mmHg at rest as proposed at the Sixth World Symposium on Pulmonary Hypertension [2]. The European Society of Cardiology (ESC) and European Respiratory Society (ERS) have reflected this in their new diagnostic criteria [1^▪▪^]. The ESC/ERS guidelines outline the different phenotypes of pulmonary hypertension based on the underlying cause as follows:

(1) Group 1 – pulmonary arterial hypertension (PAH)
(2) Group 2 – pulmonary hypertension associated with left heart disease (PH-LHD)
(3) Group 3 – pulmonary hypertension associated with lung disease (PH-LD) and/or hypoxia
(4) Group 4 – pulmonary hypertension associated with chronic pulmonary artery obstructions
(5) Group 5 – pulmonary hypertension with unclear and/or multifactorial mechanisms

PAH is most commonly idiopathic but can be the result of connective tissue diseases, congenital heart diseases (CHD), portal hypertension and drugs. PH-LHD is typically caused by diastolic heart failure, systolic heart failure or valvular disease and has the highest prevalence of all pulmonary hypertension phenotypes. PH-LD is a heterogeneous group of advanced parenchymal diseases, including interstitial lung disease (ILD), chronic obstructive pulmonary disease (COPD) and idiopathic pulmonary fibrosis (IPF). Group 4 patients are largely represented by those with chronic thromboembolic pulmonary hypertension (CTEPH). Finally, group 5 contains, but is not restricted to, systemic, haematological and metabolic disorders for which the prevalence is unknown.

The gold standard for pulmonary hypertension diagnosis is right heart catheterization (RHC) but a multimodal diagnostic approach, including CT, is required to phenotype and risk assess patients [1^▪▪^].

## COMPUTER TOMOGRAPHY IN PULMONARY HYPERTENSION

CT imaging provides information on the pathophysiological impact of pulmonary hypertension on cardiothoracic structures, allowing clinicians to understand the underlying phenotype and target therapies accordingly. ESC and ERS recommend that CT imaging is performed in all patients with suspected pulmonary hypertension [1^▪▪^,^3^]. This allows for optimal imaging of the lung parenchyma and a gross evaluation of cardiac structures and the pulmonary vasculature. CT pulmonary angiography (CT with iodinated contrast in the pulmonary arterial phase) is required in patients with suspected CTEPH. The Pulmonary Vascular Research Institute imaging statement [3] places CT pulmonary angiography more centrally in the diagnostic pathway for all pulmonary hypertension patients given the additional diagnostic value for chronic embolic disease, vascular anomalies and an assessment of the cardiac structures. Although noncontrast CT imaging can provide valuable information of parenchymal disease in those with suspected PH-LD, contrast-enhanced CT, specifically CT pulmonary angiography, is recommended for a more complete imaging assessment, irrespective of the suspected pulmonary hypertension aetiology.

**FIGURE 1:**
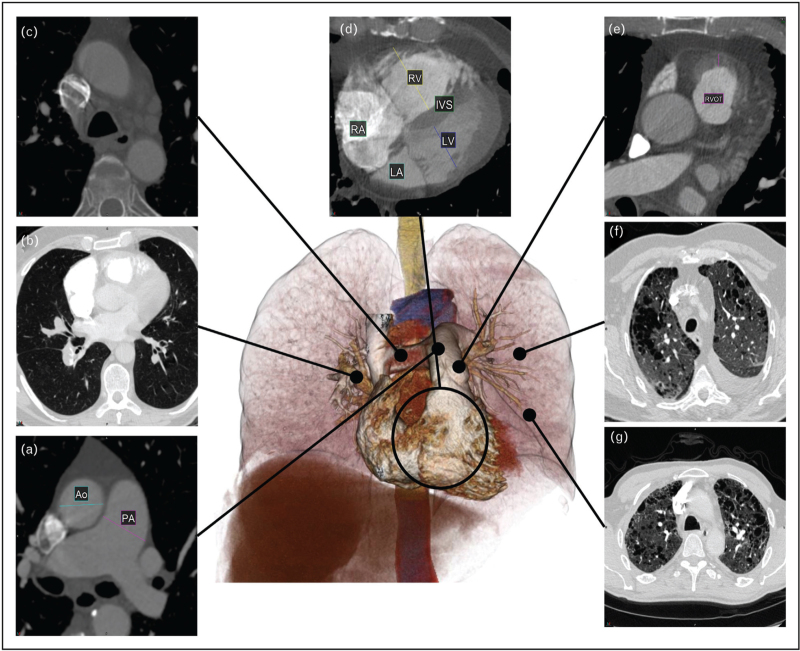
Computer tomography findings in pulmonary hypertension. (a) Dilatation of the main pulmonary artery. Comparison to the aorta is used to normalize for body size. (b) The pulmonary arteries are assessed for thromboembolic disease and pulmonary obstruction. These provide information on AV malformations, aneurysms, and large vessel vasculitis. (c) Mediastinal structures including the dilated bronchial arteries, dilated oesophagus, lymphadenopathy and pericardial effusions provide evidence of pulmonary hypertension and potential cause. (d) Size and shape of cardiac chambers and myocardial hypertrophy provide evidence of left-sided and right-sided heart failure. Assessment of congenital heart disease and anomalous arterial venous drainage can also be made. (e) Thickening of the right ventricle (RV) outflow tract is suggestive of RV hypertrophy. (f) Other thoracic evidence such as pleural effusion, pericardial effusion and ascites and features of left and right heart failure should be considered. (g) Lung parenchymal assessment provides information as to the presence and severity of lung disease as important factors for differentiation of pulmonary hypertension phenotypes.

Features of pulmonary hypertension on CTPA imaging [4–6] are presented in Fig. 1. Dilatation of the main pulmonary artery is seen in isolation and in comparison to the aorta (Fig. 1a) with evidence suggesting that a pulmonary artery diameter greater than 30 mm is sensitive and specific for pulmonary hypertension [7]. The pulmonary arteries are further assessed for pulmonary emboli (Fig. 1b), indicating a group 4 phenotype. Arteriovenous (AV) malformations, aneurysms, and large vessel vasculitis may also be present suggesting a group 1, PAH phenotype. Dilated bronchial arteries suggest chronic pulmonary vascular disease, typically chronic embolic disease causing regional pulmonary arterial hypoxaemia (Fig. 1c).

Morphology of the cardiac chambers and myocardial hypertrophy provide evidence of left-sided and right-sided disease manifestations in pulmonary hypertension. Right ventricular hypertrophy, deviation of the interventricular septum, and pulmonary artery dilatation are the three features that indicate increased pulmonary arterial pressures (Fig. 1d). Right ventricular hypertrophy is commonly assessed at the right ventricular outflow tract (given the compacted nature at this anatomical location), with thickness ≥6 mm suggesting pulmonary hypertension (Fig. 1e). CHD and anomalous arterial venous drainage can be visualized [4]. Other nonspecific features of heart failure may be present such as pericardial or pleural effusions (Fig. 1f) [1^▪▪^].

Lung parenchymal assessment provides information as to the type and severity of lung disease. Increases in lung density (or attenuation) are seen in ground glass opacification or consolidation. Density reductions are seen in emphysema-related lung destruction and mild reductions are seen in mosaic perfusion abnormalities or air trapping. The distribution should be assessed with specific diseases, such as ILD, presenting with characteristic patterns like honeycombing and reticulation (Fig. 1g). In pulmonary hypertension, overlapping imaging patterns emerge [5], highlighting the need for robust modes of parenchymal assessment to phenotype those with pulmonary hypertension.

## ARTIFICIAL INTELLIGENCE IN PULMONARY HYPERTENSION

Artificial intelligence involves programming computers to perform tasks that typically require human intelligence. Machine learning, a subset of artificial intelligence, allows computers to learn and recognize patterns or features from data without the need for explicit programming. Deep learning is a form of machine learning that utilizes many layers, an example being convolutional neural networks (CNN). CNNs are widely regarded as effective techniques in computer vision for tasks such as feature recognition, localization, and classification [8–11]. These networks feed into radiomic applications such as segmentation, texture analysis, and classification, which will be explored subsequently.

### Cardiac segmentation

**FIGURE 2:**
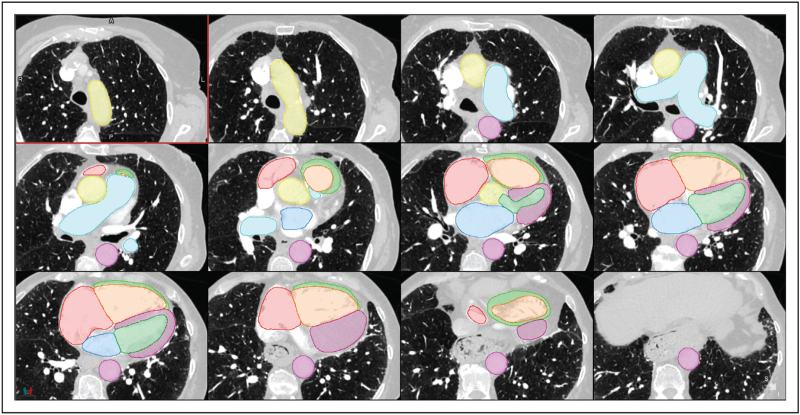
Cardiac and great vessel segmentation. Segmentation of the cardiac structures and great vessels including ascending and descending aorta (yellow and pink, respectively), pulmonary artery (light blue), right atrium (red), right ventricular chamber (orange) and myocardium (light green), left atrium (dark blue), left ventricular chamber (dark green) and myocardium (purple). For a colour version of this figure, see the online version of this article.

Semantic segmentation in medical imaging is the classification and delineation of pixels/voxels into regions representing anatomical structures. This identifies and quantifies morphological features such as cardiac chamber masses, volumes and vessel diameters. These parameters are crucial for disease diagnosis, treatment planning, prognostication and disease monitoring [12–15]. Manual segmentation is time-consuming [12,16] with significant variability [17,18^▪^,^19^]. Automatic segmentation has evolved with the invention of deep learning to match human performance (Fig. 2). UNet is a state-of-the-art CNN widely used for segmentation that excels in image segmentation by using an encoder–decoder architecture to efficiently capture context and spatial information [20–22].

Segmentation performance is evaluated using a combination of area overlap metrics and surface boundary distance metrics. The dice similarity coefficient (DSC) is a widely used overlap metric, with scores ranging from 0 to 1 representing no or perfect overlap between the artificial intelligence-generated segmentation and the ground truth, respectively. Surface distance metrics, such as Hausdorff95 or normalized surface distance, measure how closely the surfaces of the segmented regions match [23^▪^].

Segmentation tools have been developed to identify individual structures for specific diagnostic purposes. Yuan *et al.*[24] introduce PA-Net, a 2D network designed for pulmonary artery segmentation in CTPA images for pulmonary embolism diagnosis. This demonstrated improved accuracy versus other state-of-the-art segmentation tools with a DSC score of 0.938 compared with the manual segmentation.

Another structure of high clinical importance is the left atrium. Its volume, when indexed by body surface area (LAVI), is a surrogate marker for chronically raised left ventricular diastolic pressure [25], found in PH-LHD, and is associated with raised all-cause mortality [26]. Aquino *et al.*[27^▪^] propose a method for left atrium segmentation in multiphase cardiac CTs, utilizing a 3D image-to-image network with a conditional variational autoencoder (cAVE). Tested on 55 patients awaiting ablation for atrial fibrillation with CT coronary angiogram, the hybrid network generates multiple segmentations with the cAVE identifying plausible segmentation distributions. The artificial intelligence-derived left atrial volume was comparable with manual measurements and was completed in less than half the time.

Sharkey *et al.*[18^▪^] developed a multistructure segmentation tool, employing a two-stage localization and segmentation methodology using nnU-Net [21] to segment nine cardiac structures on CTPA imaging. Trained on 100 patients with suspected pulmonary hypertension, the model demonstrates DSC scores greater than 0.85 for four-chamber, aorta and pulmonary artery structures. DSC for RV hypertrophy was lower for RV hypertrophy 0.58 correlating with lower interobserver agreement of the radiologists measurements of this structure. Visual assessment was conducted in 1333 patients with suspected pulmonary hypertension or suspected pulmonary embolism with no difference in performance between the two patient cohorts, increasing applicability across different pulmonary hypertension phenotypes [18^▪^]. To our knowledge, this is the only multistructure segmentation tool, trained in a pulmonary hypertension cohort.

Chen *et al.*[28] extend the segmentation to 19 cardiac substructures in a lung cancer cohort using the same nnU-Net. They employ separate models for different substructure groups, balancing memory requirements and potentially enhancing inference speed. The segmentation achieves a high mean DSC score with 94% of contours deemed clinically acceptable for radiotherapy treatment planning.

A potential challenge is handling complex anatomy like CHD, and severe cardiac and vascular disease manifestations in PAH, utilizing 2D and 3D UNets, Yao *et al.*[12] uniquely conduct cardiac structure segmentation across 14 types of CHD. They segment chambers and myocardium at low resolution and blood pool at high resolution before combining them. Graph theory is then applied for patient-specific heart and vessel graph generation, improving vessel categorization in complex CHD. Compared with the previous state-of-the-art method, this segmentation enhances DSC scores by 12% [29].

### Lung parenchymal assessment

Accurate lung parenchymal assessment is vital to phenotype pulmonary hypertension with significant crossover between different groups, especially group 1 PAH and group 3 PH-LD [4]. Historically quantitative evaluation of the lung parenchyma was made based on density assessment, more recently artificial intelligence tools have been developed using various radiomic methods such as texture analysis and classification, which are often used together. The outputs contribute to diagnosis and quantification of disease.

**FIGURE 3:**
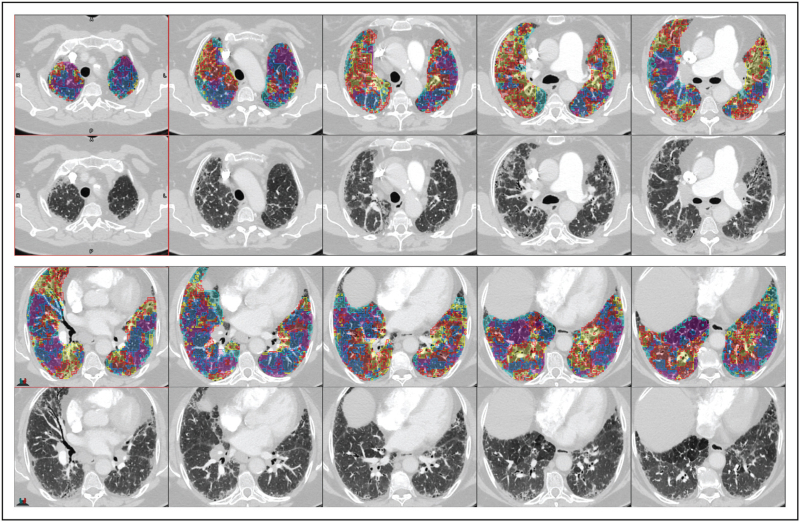
Lung disease texture quantification and localization. Patient with interstitial lung disease overlaid with artificial intelligence lung disease texture classifications; normal (dark blue), pure ground-glass (red), ground-glass with reticulation (yellow), honeycombing (light blue) and low attenuation (pink). For a colour version of this figure, see the online version of this article.

Texture analysis, in the context of deep learning, is the characterization of different regions of an image based on the local pixel intensities. The model learns its own set of filters, or weights, to identify differing textures (or tissues) in the training set. The identified regions of abnormal and normal tissue create an overlay map of the lung, enabling localization and quantification of disease (Fig. 3). Classification is the use of deep learning to identify patterns of disease to make single or multiclass predictions. Classification methods are typically used for automated diagnosis but can be used in prognostication.

These techniques, when used in isolation, have defined utility. Touloumes *et al.* introduced a CNN classification network for pulmonary fibrosis diagnosis. Trained on 3600 CT scans and fine-tuned in an external US cohort, the model had an AUC of 0.997 [30] with high sensitivity and specificity (91.3 and 95.3%) in both low and high pulmonary fibrosis prevalence populations [31^▪^]. However, this model does not directly quantify disease burden, a limitation of classification models.

To provide prognostic insight from a classification model, Mei *et al.*[32^▪^] used a CNN pretrained on RadImageNet to classify five ILD subtypes and further predict 3-year survival with a transformer model. For prognostication, they used a novel time-series multimodal model, integrating clinical, medication, and imaging data, to enhance survival predictions. The study further demonstrated that using this multimodal data improved classification predictions. This suggests longitudinal, holistic use of data to generate artificial intelligence models to improve diagnostics and prognostication.

To comprehensively assess lung parenchyma, Sharkey *et al.*[33^▪^] developed and tested an artificial intelligence tool using segmentation, texture analysis, and classification techniques on 122 patients with RHC confirmed pulmonary hypertension. They used nnU-Net to first segment the lung volume from CTPA images. This had a DSC score of 0.99 in the internal cohort and was shown to be effective in an external cohort with one failure out of 28 tested [34]. They then classified and quantified five different parenchymal patterns in a combined cohort of idiopathic PAH and PH-LD using a patch-based DenseNet-121 classification model. The proportion of each lung texture was calculated to determine disease severity. Area under the receiver-operating characteristics curve (AUC) was 0.94 and 0.95 for internal and external test sets, respectively. The model showed strong correlation with diffusing capacity of carbon monoxide (DLCO), and good correspondence with disease severity reported by specialist radiologists [33^▪^]. This suggests utility in disease identification, quantification and lung function assessment. The clinical impact was consolidated with the finding that artificial intelligence-quantified percentage of fibrosis is an independent mortality predictor in patients with PAH or PH-LD [35^▪^].

Handa *et al.*[36] developed a similar artificial intelligence tool in the context of IPF. They trained the artificial intelligence on 304 High-Resolution CT (HRCT) images for patients with diffuse lung disease. They successfully quantified 10 parenchymal lung patterns plus airway volumes, achieving DSC scores of 0.67, 0.76, and 0.64 versus visual scoring for reticulation, honeycombing, and bronchi, respectively. In 120 IPF patients, over a median follow-up of 2184 days, the artificial intelligence-measured lung and bronchial volumes were found to be prognostic with hazard ratios of 0.97 and 1.33, respectively.

### Pulmonary arterial clot detection

Approximately 2–3% of acute pulmonary embolisms develop into CTEPH [37^▪^,^38^] and with interventions such as PEA available for those with chronic thromboembolic disease (CTED) [1^▪▪^], identification of both acute and chronic pulmonary embolism is a clinically relevant endeavour.

There is a strong literature base for the automated detection of acute pulmonary embolism as deep learning reading of CTPA could enable automatic worklist prioritization, quantification and characterization of disease, and smart reporting [38,39]. A recent systematic review of deep learning for pulmonary embolism detection [39] found five studies prior to 2021 with pooled sensitivity and specificity for pulmonary embolism detection of 0.88 [95% confidence interval (CI) 0.803–0.927] and 0.86 (95% CI 0.756–0.924), respectively. All studies analysed utilized a CNN to analyse the imaging data, with one study additionally including clinical data available in the electronic health record. The algorithms were very sensitive but despite the implementation of deep learning, still had high false-positive rate.

More recently, multiple studies have assessed the performance of an artificial intelligence pulmonary embolism detection system (AIDOC v1.0, AIDOC Medical, Tel Aviv, Israel), which is both CE and Food and Drug Administration (FDA)-approved. Cheikh *et al.*[40^▪^], in a cohort of 1202 patients with a 15.8% prevalence, revealed that the artificial intelligence system had greater sensitivity (92.6%) and negative-predictive value (NPV, 98.6%) compared with the radiologists (90% sensitivity and 98.1% NPV). The radiologists had greater specificity (99.1%) and positive-predictive value (PPV, 95%) versus the artificial intelligence algorithm (95.8% specificity and 80.4% PPV). The artificial intelligence detected an additional 19 pulmonary embolisms. The high diagnostic accuracy was corroborated in subsequent research [41,42]. This suggests a combination of artificial intelligence and radiologists could enhance clinical practice, with radiologists reducing artificial intelligence's overcall and artificial intelligence detecting pulmonary embolisms that might be missed otherwise.

The literature on artificial intelligence detection of chronic pulmonary embolism (CPE) is sparse in comparison to artificial intelligence detection of acute pulmonary embolism. Ma *et al.*[43] introduced a method to predict pulmonary embolism presence, location (left/right/central), and condition (acute/chronic). CPE detection was significantly lower than acute with AUCs of 0.69 and 0.89–0.95, respectively, highlighting the challenge of accurately diagnosing CPE.

Vainio *et al.*[44^▪^] introduced a novel methodology, utilizing 11 2D maximum intensity projection (MIP) images of volumetric CT scans to identify CPE. A multinetwork ensemble model achieves a classification AUC of 0.94 in a local dataset. Left and right lungs were processed separately, with nonlung tissue removed through lung segmentation. Although this approach compels the network to focus on the lung vasculature because of limited additional information, MIP creation leads to a significant loss of information about mosaicism, often used by radiologists in CPE detection.

## DEVELOPMENT IN IMAGING ACQUISITION

Artificial intelligence tools are yet to take advantage of novel imaging techniques such as dual-energy CT (DECT), lung subtraction iodine mapping (CT-LSIM) or high-resolution imaging with photon-counting CT.

DECT provides distinct images for differing tissue types by modulating the x-ray energy or spectrum. This improves image quality, increasing diagnostic confidence with widespread applications in cardiothoracic imaging [45,46]. However, because of previous issues with increased noise and imaging acquisition times, this technology did not have widespread adoption. Advancements in this technology has increased uptake and offers a novel imaging modality for artificial intelligence development [46].

CT-LSIM images are generated when noncontrast CT images are subtracted from those of contrast-enhanced CTPA. This provides high spatial resolution images of the pulmonary arterial system and parenchyma with greater specificity for pulmonary embolism detection than CT angiography. CT-LSIM has comparable diagnostic performance to DECT [47,48] without the need of dedicated hardware.

Photon-counting CT will provide a higher spatial resolution evaluation of the pulmonary vasculature, and other associated disease manifestations in pulmonary hypertension such as interstitial lung disease and chronic emboli. Utilizing artificial intelligence to support evaluation of the fine details may aid in the diagnosis and phenotyping of pulmonary hypertension in the future.

Further work is required with each imaging acquisition development to maximize the use of new spectral data or more detailed structural information.

## CONCLUSION

Various artificial intelligence tools have been applied to CT in those with pulmonary hypertension and related diseases. These demonstrate clinical utility in automated diagnosis, quantification of disease and prognostication, facilitating the phenotyping of pulmonary hypertension. Further work is required to improve the models in areas such as CPE detection. Developments in imaging acquisition techniques require parallel developments in artificial intelligence techniques to maximize use of more detailed pathophysiological data.

## Acknowledgements

*None.*

### Financial support and sponsorship

*M.J.S. was funded by the Wellcome Trust 223521/Z/21/Z. E.W.C has received no financial support or sponsorship relating to this article. A.J.S. has received no financial support or sponsorship relating to this article.*

*Disclosure of funding: this research was funded in whole, or in part, by the Wellcome Trust 223521/Z/21/Z. NIHR Sheffield Biomedical Research Centre NIHR203321. For the purpose of open access, the author has applied a CC BY public copyright licence to any Author Accepted Manuscript version arising from this submission.*

*This work was supported by an NIHR AI Award, AI_AWARD01706.*

### Conflicts of interest

*There are no conflicts of interest.*

## REFERENCES AND RECOMMENDED READING

Papers of particular interest, published within the annual period of review, have been highlighted as:

▪ of special interest
▪▪ of outstanding interest
